# Quantitative Analysis of Multicomponents in Qufeng Zhitong Capsule and Application of Network Pharmacology to Explore the Anti-Inflammatory Activity of Focused Compounds

**DOI:** 10.1155/2022/4229945

**Published:** 2022-06-29

**Authors:** Mengjie Xue, Yuting Zhao, Ying Cui, Jing Yang, Yuefei Wang, Xin Chai

**Affiliations:** ^1^State Key Laboratory of Component-based Chinese Medicine, Tianjin Key Laboratory of TCM Chemistry and Analysis, Tianjin University of Traditional Chinese Medicine, Tianjin 301617, China; ^2^Haihe Laboratory of Modern Chinese Medicine, Tianjin 301617, China

## Abstract

Qufeng Zhitong capsule (QZC) is a well-known Chinese patent medicine that has been widely applied for the clinical treatment of rheumatoid arthritis and other inflammatory diseases. To date, its material basis is still unclear, which has greatly limited its clinical application. In this study, by taking advantage of ultra-high-performance liquid chromatography tandem *Q*-Exactive Orbitrap high-resolution mass spectrometry, 16 chemical components such as gallic acid, protocatechuic acid, and neochlorogenic acid in QZC were characterized and unambiguously identified based on comparison with the corresponding reference standards. In addition, the correlation between the focused components and their corresponding raw herbs from QZC prescription was investigated. For the first time, the relationship between the components mentioned above and their anti-inflammatory activity was explored via network pharmacology analysis, and a visualized network of “medicinal materials-QZC-compounds-targets-pathways” was established. Based on the brief prediction results of network pharmacological analysis, ultra-performance liquid chromatography coupled with photodiode array detector method was validated in terms of linearity, limit of detection, limit of quantification, precision, repeatability, stability, and recovery test and was successfully employed to determine 16 compounds in 28 batches of QZCs, which confirmed the feasibility and reliability of the established method for the quantitative analysis of 16 compounds in QZC. Considering the content and bioactivity of the tested components, four compounds were recommended as candidate indicators for quality evaluation ultimately. The potential value of this study could not only support a quality evaluation of QZC but also provide a theoretical basis for further in-depth research of QZC in clinical research.

## 1. Introduction

As a clinically effective Chinese patent medicine, Qufeng Zhitong capsule (QZC), which consists of seven herbs, including Radix Angelicae Pubescentis (Duhuo, RAP), Herba Geranii (Laoguancao, HG), Radix Dipsaci (Xuduan, RD), Radix et Rhizoma Clematidis (Weilingxian, ReRC), Flos Carthami (Honghua, FC), Herba Visci (Hujisheng, HV), and Radix Aconiti Kusnezoffii Cocta (Zhicaowu, RAKc) [1], is commonly used for the treatment of inflammatory diseases owing to its good efficacy and slight side effects and drug resistance. According to the Pharmacopoeia of the People's Republic of China (ChP, 2020 Edition), QZC can be used to treat such diseases as joint swelling, limb numbness, ache of the loins and knees, and other symptoms. Rheumatoid arthritis (RA) is a chronic systemic autoimmune disease of unknown etiology, mainly characterized by erosive arthritis manifestation [2, 3]. Without early diagnosis and effective therapy, RA will eventually lead to joint deformity and loss of function [4], seriously reducing patients' quality of life and increasing their economic burden. Modern pharmacological studies have shown that QZC has anti-inflammatory, analgesic, immunomodulatory, and other pharmacological effects, which can significantly alleviate the symptoms caused by RA, such as chill, pain, swelling, and stiffness [5]. In addition, QZC can also be used for the treatment of knee osteoarthritis [6], ischemic necrosis of femoral head [7], and neuropathic pain [8].

Network pharmacology is a comprehensive analysis method based on the interaction network of disease, gene, target protein, and drug, which has been proved to be a practical way to explore the potential targets and pathways of traditional Chinese medicine (TCM) in the treatment of diseases [9]. By applying the network model to show and research the interaction between components and diseases, network pharmacology analysis explains the pharmacological mechanism of TCM prescriptions on the whole and provides a new strategy for screening the quantitative components in TCM. Therefore, network pharmacology has become an indispensable means for the development of TCM [10].

At present, the research on QZC mostly focuses on its clinical application, while much less attention has been paid to the identification and quantitative analysis of its chemical components. Moreover, studies on the underlying mechanism related to the effects of QZC are negligible because of its complex chemical components and the multi-targets networks. As is known to us, an effective quality control system is the key to ensuring the quality, safety, and efficacy of TCM in clinical use. Various chemical analysis methods such as thin-layer chromatography (TLC), high-performance liquid chromatography (HPLC), and other detection techniques have been employed for quality assessment and standardization of QZC. As the standard to evaluate the quality of QZC, ChP stipulates that the content of akebia saponin D (AsD) should not be less than 1.5 mg per capsule and gallic acid (Gaa) should not be less than 0.30 mg per capsule, which indicate that there are certain limitations such as using the limited quality indicators and the laborious operation for preparing sample solution [11, 12]. Therefore, we suggest that further research should aim to clarify the material basis and establish a comprehensive quality standard system of QZC.

In this study, 16 compounds, including iridoids, coumarins, phenolic acids, and triterpenoid saponin, were identified and traced to their herbal sources by ultra-high-performance liquid chromatography tandem *Q*-Exactive Orbitrap high-resolution mass spectrometry (UHPLC/*Q*-Orbitrap-MS) within 35 min. Besides, the relationship between the 16 compounds and their anti-inflammatory activity was investigated by network-based pharmacology analytical approaches, which provided the basis for the quantitative analysis of QZC. We also used the “spider-web” mode to optimize the sample preparation process so as to improve the efficiency of the extraction method comprehensively. For the quantitative analysis of the multi-components in QZC, a novel method was established by ultra-performance liquid chromatography with photodiode array detector (UPLC-PDA), which was validated in terms of linearity, limit of detection (LoD), limit of quantification (LoQ), precision (intra- and inter-day), repeatability, stability, and recovery test and successfully applied to the quantitative determination of 16 compounds mentioned above in 28 batches of QZCs. The results confirmed its feasibility and reliability in practice with the advantages of simple sample preparation, good chromatographic peak shape, and high repeatability.

## 2. Materials and Methods

### 2.1. Reagents and Materials

Methanol was purchased from Thermo Fisher Scientific (Fair Lawn, NJ, USA). Formic acid was purchased from Shanghai Aladdin Biochemical Technology Co., Ltd. (Shanghai, China). Water used in the experiment was purified by a Milli-*Q* water purification system (Millipore, Billerica, MA, USA).

Reference standards including Gaa, protocatechuic acid (Pra), neochlorogenic acid (Nea), chlorogenic acid (Cha), cryptochlorogenic acid (Cra), loganic acid (Loa), corilagin (Cor), loganin (Log), isochlorogenic acids A−C (IaA−C), angelol A (AnA), columbianetin acetate (Coa), osthole (Ost), columbianadin (Col), and AsD were obtained from Shanghai Yuanye Bio-Technology Co., Ltd. (Shanghai, China). The purities of these reference standards were determined to be above 98% by UPLC analysis. All the 28 batches of QZCs were provided by Shaanxi Buchang Pharmaceutical Co., Ltd. (Shaanxi, China) and numbered as S1-S28. Duhuo, Laoguancao, Xuduan, Weilingxian, Honghua, Hujisheng, and Zhicaowu were also provided by Shaanxi Buchang Pharmaceutical Co., Ltd., and identified by Prof. Yuefei Wang. All samples were deposited in the State Key Laboratory of Component-based Chinese Medicine, Tianjin University of Traditional Chinese Medicine (Tianjin, China).

### 2.2. Network Pharmacology Analysis

We followed the network pharmacology analysis methods of Xue et al. [13]. The PubChem database (https://pubchem.ncbi.nlm.nih.gov/) was used to search the structural formulas and IUPAC International Chemical Identifiers (InChI) of the tested compounds. The targets of the compounds were collected from the Swiss Target Prediction database (https://www.swisstargetprediction.ch/) and Bioinformatics Analysis Tool for Molecular Mechanism of TCM (BATMAN-TCM, https://bionet.ncpsb.org.cn/batman-tcm/) and analyzed by Ingenuity Pathway Analysis (IPA). The protein-protein interaction (PPI) data were obtained from the STRING database (https://string-db.org/). The Kyoto Encyclopedia of Genes and Genomes (KEGG) pathway enrichment analysis on target proteins was performed based on the KEGG database (https://www.kegg.jp/). Ranked by the KEGG analysis, the top 30 pathways related to inflammation were screened out and irrelevant pathways were eliminated. Origin 9.6 software was used to build a visualized network of “medicinal materials-QZC-compounds-targets-pathways.”

### 2.3. UHPLC/Q-Orbitrap-MS Analysis

The qualitative analysis was performed on a Thermo Scientific UltiMate 3000 instrument (Thermo Fisher Scientific, San Jose, CA, USA) coupled with a *Q*-Exactive Orbitrap MS equipped with heated electrospray ionization (HESI) source. Chromatographic separation was performed using an ACQUITY UPLC® BEH C18 column (2.1 × 100 mm, 1.7 *µ*m, Waters, Milford, MA, USA) at 40°C. The mobile phase system consisted of 0.1% formic acid aqueous solution (*v/v*) (A) and methanol (B) at a flow rate of 0.3 mL min^−1^ with the following gradient program: 0–5 min, 3–9% B; 5–9 min, 9–15% B; 9–11 min, 15–17% B; 11–15 min, 17–27% B; 15–18 min, 27–28% B; 18–26 min, 28–50% B; 26–31 min, 50–66% B; 31–35 min, 66–74% B; 35–36 min, 74–3% B. The injection volume for all samples was 2 *μ*L. The MS method used in this study was adopted from Xue et al. [13]. The mass spectrometer was carried out in both positive and negative ion modes, which was operated in Full MS and dd-MS^2^ (TopN) modes simultaneously. Ions were scanned at high resolution (70000 in MS^1^, 17500 in MS^2^), and the MS scan range was 100–1500 *m/z* at both MS^1^ and MS^2^ levels. The maximum injection time of 50 and 100 ms was used for MS^1^ and MS^2^, respectively. The automatic gain control (AGC) target was set to 3e^6^ and 1e^5^ in MS^1^ and MS^2^, respectively. The optimal MS parameters were as follows: spray voltage, −2.5 kV (in negative ion mode), 3.5 kV (in positive ion mode); sheath gas flow rate, 35 Arb; aux gas flow rate, 10 Arb; capillary temperature, 350°C; aux gas heater temperature, 350°C. All data were acquired and processed using the Thermo Scientific™ Xcalibur™ system.

### 2.4. UPLC-PDA Conditions for Quantitative Analysis

Equipped with a binary solvent manager, an autosampler, and a PDA detector, an ACQUITY UPLC H-class PLUS system (Waters) was used to perform the quantitative analysis. A multiwavelength switching method was adopted on the PDA detector, in which the detection wavelength was set as follows: 0.00–2.20 min at 300 nm, 2.20–3.50 min at 259 nm, 3.50–5.20 min at 300 nm, 5.20–18.00 min at 239 nm, and 18.00–35.00 min at 325 nm. The wavelength was set at 211 nm separately for the detection of AsD in consideration of its maximum response at 211 nm. Other liquid chromatography parameters were the same as the qualitative analysis described above.

### 2.5. Standard Solution Preparation

A total of 16 reference standards were accurately weighed and dissolved in 10–50% methanol aqueous solutions (*v/v*) to prepare stock solutions, respectively. A certain amount of each stock solution was placed in a 10 mL volumetric flask and diluted to volume with 10% methanol aqueous solution at the concentration of 24.89 *μ*g mL^−1^ Gaa, 1.332 *μ*g mL^−1^ Pra, 3.034 *μ*g mL^−1^ Nea, 7.442 *μ*g mL^−1^ Cha, 3.036 *μ*g mL^−1^ Cra, 33.92 *μ*g mL^−1^ Loa, 10.68 *μ*g mL^−1^ Cor, 7.686 *μ*g mL^−1^ Log, 2.490 *μ*g mL^−1^ IaB, 1.747 *μ*g mL^−1^ IaA, 3.192 *μ*g mL^−1^ IaC, 9.231 *μ*g mL^−1^ AnA, 6.269 *μ*g mL^−1^ Coa, 10.86 *μ*g mL^−1^ Ost, 1.602 *μ*g mL^−1^ Col, and 138.2 *μ*g mL^−1^ AsD. The mixed standard solution was diluted stepwise with 50% methanol solution to obtain seven different concentrations for plotting the calibration curves. All the standard solutions were stored at 4°C when not in use.

### 2.6. Sample Solution Preparation

QZC powder (0.5 g) precisely weighed was transferred into a 25 mL volumetric flask and then ultrasonically extracted with 75% methanol aqueous solution at 60°C for 30 min. After cooling down to room temperature, the extracted solution was diluted to scale by 75% methanol aqueous solution and centrifuged at 12700 rpm for 10 min.

Weilingxian and Duhuo were pulverized into homogeneous powder and about 0.5 g of the above powder was weighted accurately and then extracted with ultrasonic assistance in 25 mL of 75% methanol aqueous solution at 60°C for 30 min separately. The solutions were centrifuged at 12700 rpm for 10 min. Honghua, Laoguancao, Hujisheng, Zhicaowu, and Xuduan were refluxed twice with water for 3 h each time, respectively, and the filtrates were subsequently centrifuged at 12700 rpm for 10 min.

QZC, Weilingxian, and Duhuo solutions were diluted 25 times with water for UHPLC-MS/MS analysis, respectively, while Honghua, Laoguancao, Hujisheng, Zhicaowu, and Xuduan solutions were diluted 25 times with 10% methanol aqueous solution for UHPLC-MS/MS analysis, respectively. QZC solution was diluted twice with water for UPLC-PDA analysis.

### 2.7. Method Validation of UPLC-PDA Analysis

The calibration curves were plotted with the concentration of tested reference as the *x*-axis and the peak area as the *y*-axis. The LoD and LoQ were measured as concentrations corresponding to a signal-to-noise ratio of 3 : 1 and 10 : 1, respectively. The intra- and inter-day precisions were carried out by six repetitive injections on the same day and for three consecutive days. The stability test was evaluated by injecting the same sample solution at 0, 2, 4, 6, 8, 10, and 12 h after preparation, respectively. The repeatability was determined by analyzing six prepared samples from the same source. The recovery was investigated by adding an accurate amount of standard solutions to 0.25 g sample powder. Six samples were prepared in parallel according to the preparation method of sample solution.

### 2.8. Data Analysis

The heat map, box plot, and parallel coordinate plot were performed using Origin 2019 software (OriginLab Ltd., Northampton, MA, USA).

## 3. Results and Discussion

### 3.1. Characterization of Chemical Constituents from QZC and Explore Drug-Disease Correlation with Network Pharmacology Strategy

A UHPLC/*Q*-Orbitrap-MS method was established to characterize the chemical constituents in QZC. As a result, sixteen compounds, including two iridoids, four coumarins, nine phenolic acids, and one triterpenoid saponin, were identified by comparing the retention times and mass spectrometry data with reference standards. Then, the established method was applied to detect components in raw herbs from the QZC prescription to trace the herbal sources of the identified components. The information of all the compounds is summarized in Table 1, the chemical structures of which are shown in Figure S1.

Based on computational biology, network analysis, and other disciplines, network pharmacology can reveal the complex network relationship among drugs, targets, and diseases so as to display the network of drugs-compounds-targets-pathways visually [14, 15]. Given the multi-components and multi-targets characteristics of TCM, network pharmacology is often used to explore the scientific mechanism and potential pharmacological activity of TCM due to its characteristics of integrity, systematicness, and comprehensiveness [16, 17].

**Figure 1:**
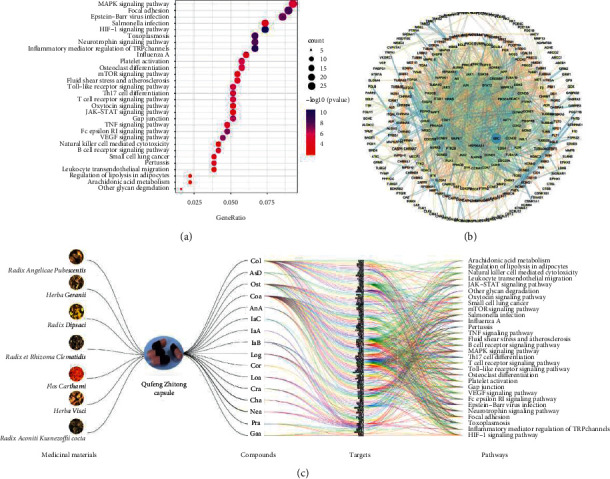
Network pharmacological analysis of QZC: (a) KEGG network diagram, (b) PPI network, and (c) parallel coordinate plot for the medicinal materials-QZC-compounds-targets-pathways.

Zhang et al. found that QZC could upregulate the level of inflammatory cytokine IL-10, inhibit synovial hyperplasia, and reduce cartilage damage in rats with arthritis [18]. Relevant pharmacological studies have shown that 10–50% of patients with RA also suffer from pulmonary fibrosis. QZC could inhibit the expression of pulmonary fibrosis-related inflammatory factors such as IL-6, IFN-*γ*, TNF-*α*, and IL-17A to ease alveolitis and pulmonary fibrosis in the bleomycin-induced mouse pulmonary fibrosis model [19]. In addition, QZC can also be applied to treat various inflammatory diseases, such as osteoarthritis and frozen shoulder [20, 21]. Therefore, in the present study, we employed network pharmacology to explore the correlation between the 16 identified compounds and their anti-inflammatory activity. Obtained from the Swiss Target Prediction database and BATMAN-TCM, the candidate targets with prediction scores of more than 3.35 were selected as the potential targets of QZC. After eliminating the duplicates, 415 targets were screened, and 223 pathways were obtained from the KEGG database. The top 30 pathways related to inflammation were selected, including MAPK signaling pathway, HIF-1 signaling pathway, inflammatory mediator regulation of TRP channels, and other pathways, and 109 corresponding targets were obtained. The KEGG analysis, PPI analysis, and visualized network of “medicinal materials-QZC-compounds-targets-pathways” are shown in Figure 1. The targets corresponding to the top 30 pathways are shown in Table S1. IPA showed that the targets are mainly located in the cytoplasm and plasma membrane. According to PPI analysis, a total of 414 nodes and 1021 edges were displayed in the PPI network with PPI-enrichment *P* value <1.0e −16. These results indicated that all the 16 compounds exhibit potential anti-inflammatory pharmacological activity.

### 3.2. Optimization of Sample Preparation and Chromatographic Conditions

Extraction variables, including extraction solvent (25%, 50%, 75%, and 100% methanol aqueous solution), ratio of material/solvent (1 : 25, 1 : 50, and 1 : 125 g mL^−1^), extraction time (20, 30, and 40 min), and extraction temperature (25, 40, 50, and 60°C) were investigated by single-factor experiment to obtain higher extraction efficiency of the tested compounds. To show the optimization process of the extraction method intuitively, the “spider-web” mode proposed by our research group in 2016 [22, 23] was applied to comprehensively analyze the effects of different extraction conditions on the extraction efficiency of all the tested compounds, such as Gaa, Pra, Nea, Cha, Cra, Loa, Cor, Log, IaB, IaA, IaC, AnA, Coa, AsD, Ost, and Col. The content of the target compounds was assigned as *C*_*m-k*_; the maximum content value of each compound in the tested extracting conditions was assigned as *C*_*k*(max)_; the normalized value of content was assigned as *E*_*m-k*_. Among them, *m* was denoted as different extract condition, namely extraction solvent (s25, s50, s75, and s100), ratio of material/solvent (r25, r50, and r125), extraction time (t20, t30, and t40), and extraction temperature (T25, T40, T50, and T60). *K* stood for the different compounds, namely Gaa, Pra, Nea, Cha, Cra, Loa, Cor, Log, IaB, IaA, IaC, AnA, Coa, AsD, Ost, and Col. The calculation formula is shown in

(1) $$\begin{array}{c} E_{m-k}=\frac{C_{m-k}}{C_{k\left(\max\right)}}. \end{array}$$

Using the extracting method at 40°C as an example, *E*_*T*40−Gaa_, *E*_*T*40−Pra_, *E*_*T*40−Nea_, *E*_*T*40−Cha_, *E*_*T*40−Cra_, *E*_*T*40−Loa_, *E*_*T*40−Cor_, *E*_*T*40−Log_, *E*_*T*40−IaB_, *E*_*T*40−IaA_, *E*_*T*40−IaC_, *E*_*T*40−AnA_, *E*_*T*40−Coa_, *E*_*T*40−AsD_, *E*_*T*40−Ost_, and *E*_*T*40−Col_ were used to set up sixteen dimensions (*P*_*i*_) of the “spider-web” mode. *α*, the angle between two adjacent dimensions, was 22.5°. By calculating the regression area of the “spider-web,” the efficiency of extracting index compounds was comprehensively evaluated. The regression area of the “spider-web” was marked as *S* and calculated to be 2.78. The calculation formula is shown in (2).

**Figure 2:**
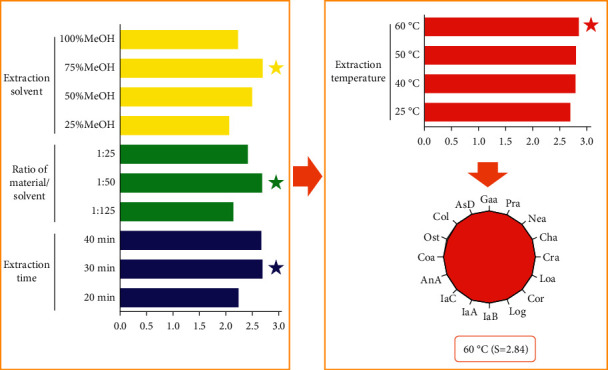
Optimization of extraction conditions by “spider-web” for QZC.

Based on the principle that the larger the regression area of the “spider-web,” the higher the extraction efficiency of the compounds, we proved that 75% methanol aqueous solution for 30 min at a ratio of 1 : 50 material/solvent by heating (60°C) is the optimal extraction condition with a “spider-web” area at 2.84, which is shown in Figure 2.

(2) $$\begin{array}{c} S=\frac{1}{2}\sin\;\alpha \left(\overset{n-1}{\underset{i=1}{\sum }}P_{i}\times P_{i+1}+P_{n}\times P_{1}\right). \end{array}$$

The chromatographic conditions were optimized to accurately determine the 16 chemical components in QZC with satisfactory peak shape, sensitivity, and resolution. Adding 0.1% formic acid to the mobile phase helped achieve satisfactory peak symmetry, and methanol was chosen to enhance sensitivity and resolution. Therefore, a gradient elution system composed of 0.1% formic acid-methanol was chosen. The column temperature was set at 40°C, improving the separation and peak shape. To attain lower noise and satisfactory sensitivity, we established a UV wavelength switching method using a variable-wavelength detector and four wavelengths, including 239, 259, 300, and 325 nm, were selected as the detective wavelengths for determining the different compounds in QZC.

### 3.3. Method Validation of UPLC-PDA Analysis for Quantitation of 16 Compounds in QZC

**Figure 3:**
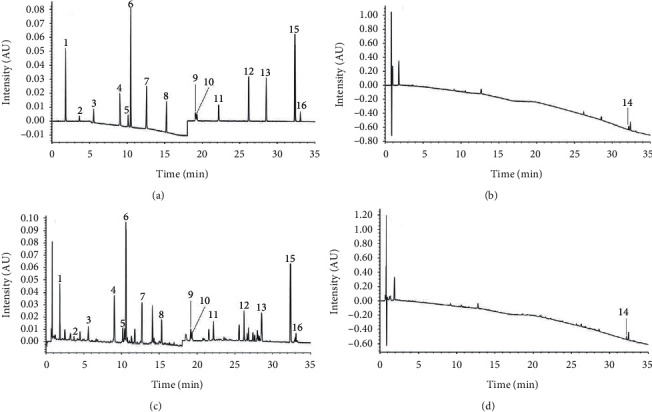
UPLC-PDA chromatograms: (a) the mixed standard solution acquired by wavelength switching mode; (b) AsD in the mixed standard solution at 211 nm; (c) QZC sample solution acquired by wavelength switching mode; (d) AsD in QZC sample solution at 211 nm. 1: Gaa; 2: Pra; 3: Nea; 4: Cha; 5: Cra; 6: Loa; 7: Cor; 8: Log; 9: IaB; 10: IaA; 11: IaC; 12: AnA; 13: Coa; 14: AsD; 15: Ost; 16: Col.

Up to now, there have been limited studies on quantitative analysis of chemical compounds in QZC, especially the multi-components quantitative analysis. Therefore, a method for the simultaneous determination of 16 compounds in QZC by UPLC-PDA was established for the first time. In addition, the UV wavelength switching method was employed, which made the 16 compounds have satisfactory peak shape, sensitivity, and resolution. The typical chromatograms of sample and mixed standard solutions are shown in Figure 3. The methods of validation, including linear regression, LoD and LoQ, precision, repeatability, stability, and recovery for 16 compounds, are summarized in Table 2. All of the calibration curves showed good linearity over the tested range with determination coefficient (*R*^*2*^) values greater than 0.999, which was qualified to be used for quantitative analysis of the focused compounds in QZC. The LoD and LoQ values were 0.02118–1.728 *μ*g mL^−1^ and 0.08707–3.455 *μ*g mL^−1^ for the 16 compounds quantified in this study, respectively. The RSD values of the measured intra- and inter-day precisions were lower than 2.6% and 2.9%, respectively, indicating that the instrument has good precision. The experimental method had good repeatability with RSD values below 2.9% and the RSD values of the sample stability test were lower than 2.4%, showing that the sample solution could remain stable within 12 h. In addition, the recovery rates for spiked samples ranged from 92.1% to 108.2%. In summary, all the results of the experiments were within an acceptable range of quantitative research, which demonstrated that the established method is rapid and simple with good accuracy and reproducibility for the content determination of 16 compounds in different batches of QZCs. Compared to the previously reported analytical methods [8, 11, 12], the established method in our study exhibited the advantages of shorter analytical time, higher resolution, and more tested compounds, which provides a better alternative for evaluating the quality of QZC.

### 3.4. Visual Modes of the Quantitative Analysis Results of 16 Components in Different Batches of QZCs

**Figure 4:**
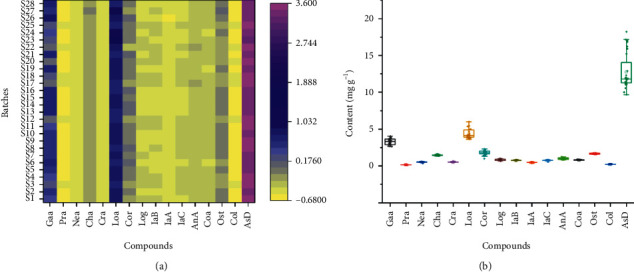
Heat map and box plot of 16 compounds in 28 batches of QZCs.

The validated UPLC-PDA method was subsequently used to determine the content of 16 compounds in 28 batches of QZCs. As shown in Table S2, the mean total content of the detected compounds is 31.44 mg·g^−1^, the detailed content of which is listed as follows: Gaa between 2.644 and 4.049 mg·g^−1^, Pra between 0.1175 and 0.1846 mg·g^−1^, Nea between 0.3662 and 0.5921 mg·g^−1^, Cha between 1.354 and 1.609 mg·g^−1^, Cra between 0.4363 and 0.6689 mg·g^−1^, Loa between 3.640 and 6.008 mg·g^−1^, Cor between 0.9905 and 2.316 mg·g^−1^, Log between 0.6843 and 1.003 mg·g^−1^, IaB between 0.7053 and 0.8372 mg·g^−1^, IaA between 0.3942 and 0.5321 mg·g^−1^, IaC between 0.6495 and 0.8388 mg·g^−1^, AnA between 0.8528 and 1.245 mg·g^−1^, Coa between 0.7487 and 0.8916 mg·g^−1^, AsD between 9.652 and 18.24 mg·g^−1^, Ost between 1.567 and 1.777 mg·g^−1^, and Col between 0.2017 and 0.2359 mg·g^−1^, respectively. The RSD values of the different detected components ranged from 4.2% to 19.1%, showing that the content of 16 compounds in different batches of QZCs varied to a certain extent. And fluctuation in the content of the tested compounds may due to the fact that the raw materials come from different sources. In order to intuitively display the trend of content distribution, we adopted a heat map and box plot, which are shown in Figure 4. The heat map reflects the fluctuation of 16 compounds in different batches through the gradient colour. Among the 16 index components, the colour depths of Gaa, Cor, and AsD fluctuate obviously, showing great differences in the content of the tested compounds from different batches. The box size represents the dispersion degree of 16 index components among different batches, among which Gaa, Loa, and AsD are relatively large. It is worth mentioning that the content of AsD is significantly higher than the other 15 components, which may due to its high content (>2%) in Xuduan stipulated in ChP. More attention should be paid to other high-content components such as Gaa, Cha, Loa, Ost, and Cor in the future research of QZC.

Required by ChP, the quality of QZC is evaluated primarily by detecting the content of AsD and Gaa, proved reasonable by this study. Besides, in the current study, we also found that Cha, Loa, Cor, and Ost have potential as an evaluation index of QZC quality control. Cha, a common component contained in Xuduan, Honghua, and Hujisheng, can exert a neuroprotective effect [24]. Moreover, as a characteristic component in Xuduan, Loa can achieve an osteoprotective effect [25]. Cor is one of the major constituents in Laoguancao, which possesses antioxidant, thrombolytic, antiatherogenic, and hepatoprotective properties [26]. Ost is distributed in Duhuo with a wide range of pharmacological effects, such as osteogenic, antimicrobial, and antiviral effects [27]. As it is evident from this study, we suggest that Cha, Loa, Ost, and Cor can be used as candidate components in the further establishment of quality standards as well. The recommended candidate components cover five raw herbs of QZC, which fully conform to the principle of TCM prescription.

## 4. Conclusions

In our study, the anti-inflammatory components in QZC were mined through network pharmacology and quantified by using the UPLC-PDA analysis method for the first time. Moreover, 16 compounds extracted from QZC were identified and traced to their herbal sources by UHPLC/Q-Orbitrap-MS. The established method for simultaneous determination of 16 compounds in QZC by UPLC-PDA integrated with the UV wavelength switching method has been demonstrated to be rapid and simple with good accuracy and reproducibility, which was used to investigate the focused compounds in 28 batches of QZCs. Eventually, four compounds, Ost, Cha, Loa, and Cor, were preferentially recommended as candidate components in QZC. The results of the study could help to uncover the chemical basis of QZC and pave the way for evaluation purposes for the quality of QZC.

## Figures and Tables

**Table 1:** Characterization of chemical components from QZC by UHPLC/*Q*-Orbitrap-MS.

Peak no.	*t*_*R*_ (min)	Formula	ES^+^ (*m/z*)	ES^−^ (*m/z*)	Identification	Source
1^*∗*^	1.97	C_7_H_6_O_5_	—	169.01329	Gallic acid	HG
2^*∗*^	3.93	C_7_H_6_O_4_	—	153.0186	Protocatechuic acid	HG
3^*∗*^	5.84	C_16_H_18_O_9_	355.10245	353.0878	Neochlorogenic acid	RD, HV
4^*∗*^	9.32	C_16_H_18_O_9_	355.10239	353.08841	Chlorogenic acid	RD, FC, HV
5^*∗*^	10.63	C_16_H_18_O_9_	355.10245	353.08762	Cryptochlorogenic acid	RD, HV
6^*∗*^	10.74	C_16_H_24_O_10_	—	375.12973	Loganic acid	RD
7^*∗*^	12.92	C_27_H_22_O_18_	—	633.07214	Corilagin	HG
8^*∗*^	15.42	C_18_H_28_O_12_	—	435.15176	Loganin	RD
9^*∗*^	19.47	C_25_H_24_O_12_	—	515.11908	Isochlorogenic acid B	RD, ReRC
10^*∗*^	19.91	C_25_H_24_O_12_	—	515.11902	Isochlorogenic acid A	RD
11^*∗*^	22.6	C_25_H_24_O_12_	—	515.11896	Isochlorogenic acid C	RD
12^*∗*^	26.3	C_20_H_24_O_7_	—	377.15918	Angelol A	RAP
13^*∗*^	28.75	C_16_H_16_O_5_	289.10638	—	Columbianetin acetate	RAP
14^*∗*^	32.22	C_47_H_76_O_18_	—	973.50336	Akebia saponin D	RD
15^*∗*^	32.64	C_15_H_16_O_3_	245.11714	—	Osthole	RAP
16^*∗*^	33.08	C_19_H_20_O_5_	329.13821	—	Columbianadin	RAP

^*∗*^Compared with reference standards.

**Table 2:** Linear regression, LoD and LoQ, intra- and interday precisions, repeatability, stability, and recovery for 16 compounds.

Compound	Regression equation	*R * ^*2*^	Linear range (*μ*g mL^−1^)	LoD (*μ*g mL^−1^)	LoQ (*μ*g mL^−1^)	Precision (RSD, %)		Repeatability (RSD, %; *n* = 6)	Stability (RSD, %; *n* = 7)	Recovery (mean ± SD, %; *n* = 6)
						Intraday *n* = 6	Interday *n* = 3			
Gaa	*y* = 828748*x* − 9753	0.9998	3.111–199.1	0.1152	0.3457	0.3	1.6	1.6	0.5	96.5 ± 1.8
Pra	*y* = 1108659*x* + 303	>0.9999	0.1664–10.65	0.1664	0.3329	2.0	1.0	1.5	1.4	93.1 ± 2.4
Nea	*y* = 1453761*x* + 4194	0.9998	0.3793–24.28	0.04215	0.1264	1.7	1.2	0.9	2.4	95.3 ± 2.8
Cha	*y* = 1425838*x* + 7909	0.9998	0.9302–59.53	0.1034	0.3101	0.6	0.6	0.6	1.6	95.0 ± 1.6
Cra	*y* = 1269077*x* + 3147	0.9999	0.3795–24.29	0.1265	0.3795	2.6	2.9	0.7	2.4	97.3 ± 2.6
Loa	*y* = 1003910*x* + 8409	>0.9999	4.240–271.4	0.1570	0.4711	0.9	0.9	1.1	0.4	92.8 ± 1.5
Cor	*y* = 1418562*x* − 2143	0.9999	1.335–85.44	0.1483	0.4450	0.4	1.4	2.0	0.3	99.1 ± 1.7
Log	*y* = 1103212*x* + 2477	>0.9999	0.9607–61.48	0.1067	0.3202	0.3	1.0	2.8	0.5	97.8 ± 2.6
IaB	*y* = 1108570*x* − 504	>0.9999	0.3113–19.92	0.1038	0.3113	0.4	0.8	2.8	1.5	104.7 ± 2.6
IaA	*y* = 1359428*x* − 399	0.9999	0.2183–13.97	0.07278	0.2183	0.6	2.4	2.3	1.2	108.2 ± 1.0
IaC	*y* = 1712250*x* − 4749	0.9994	0.3990–25.54	0.1330	0.3990	1.2	2.4	2.9	1.0	106.6 ± 3.1
AnA	*y* = 1366474*x* + 2662	>0.9999	1.154–73.85	0.04274	0.1282	1.1	1.6	1.1	0.3	100.6 ± 1.1
Coa	*y* = 2007500*x* + 2657	>0.9999	0.7836–50.15	0.02118	0.08707	0.4	1.4	1.0	0.2	103.0 ± 1.2
Ost	*y* = 2326874*x* − 3601	>0.9999	1.357–86.86	0.05026	0.1508	0.4	1.0	1.0	0.1	107.6 ± 2.6
Col	*y* = 1723094*x* − 22	>0.9999	0.2003–12.82	0.06675	0.2003	1.4	2.4	1.3	1.3	102.4 ± 1.8
AsD	*y* = 118179*x* − 714	>0.9999	17.28–1106	1.728	3.455	2.4	1.9	1.4	0.6	92.1 ± 1.5

## Data Availability

The data used to support the finding of this study are available from the corresponding author upon request.

## Supplementary Materials

Supplementary Materials Figure S1: Chemical structures of 16 compounds in QZC. Table S1: The targets corresponding to the top 30 pathways. Table S2: The content of 16 compounds in 28 batches of QZCs.
