# Supplementary material for: Quantitative Analysis of Multicomponents in Qufeng Zhitong Capsule and Application of Network Pharmacology to Explore the Anti-Inflammatory Activity of Focused Compounds
Source: J Anal Methods Chem. 2022 Jun 29;2022:4229945. doi: 10.1155/2022/4229945 (PMC9259231; doi:10.1155/2022/4229945)
Supplement: Supplementary Materials — Figure S1: Chemical structures of 16 compounds in QZC. Table S1: The targets corresponding to the top 30 pathways. Table S2: The content of 16 compounds in 28 batches of QZCs. [file 4229945.f1.docx]

**Supplementary Materials to:**

**Quantitative Analysis of Multi-components in Qufeng Zhitong Capsule and Application of Network Pharmacology to Explore the Anti-inflammatory Activity of Focused Compounds**


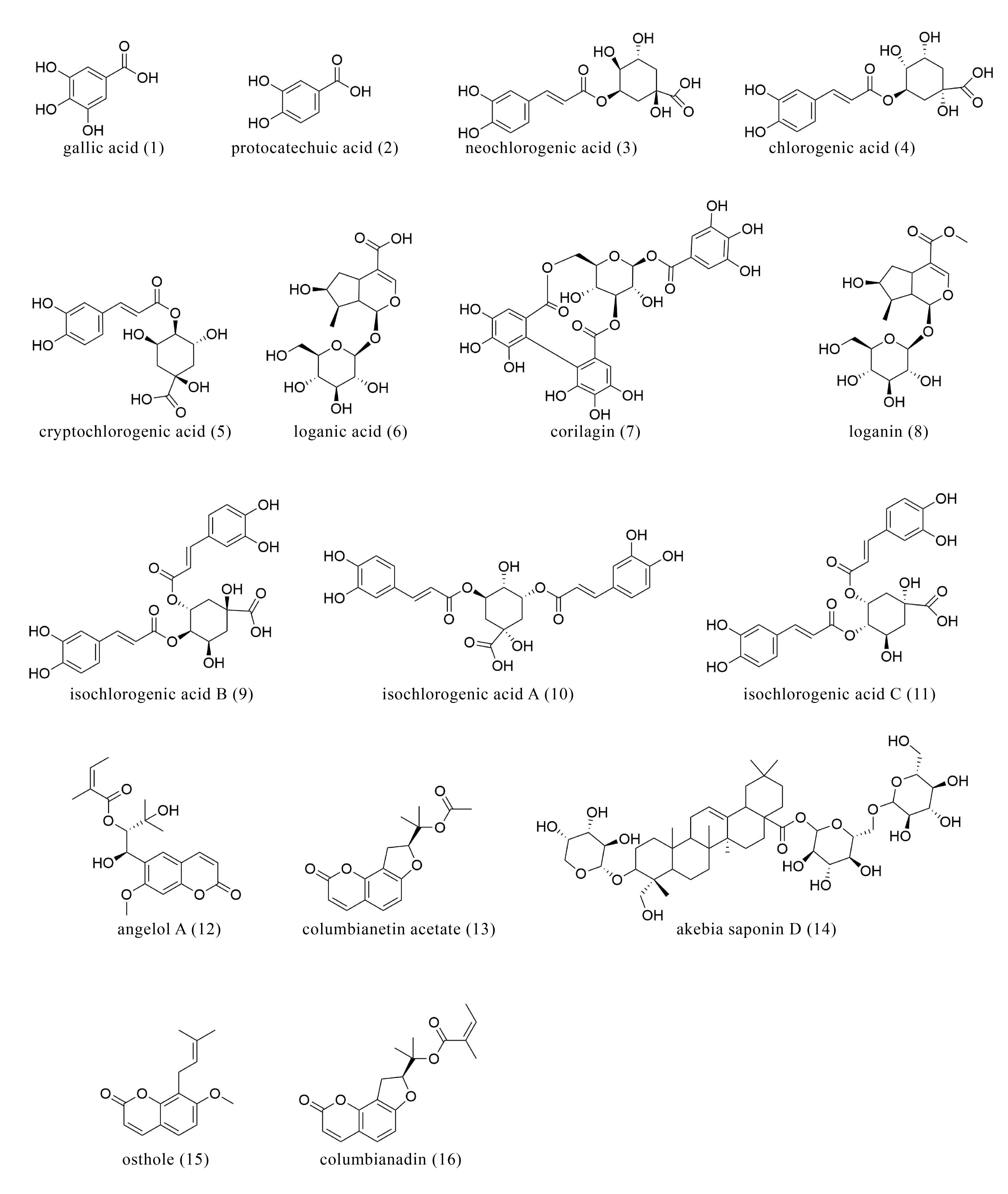


Figure S1: Chemical structures of 16 compounds in QZC

Table S1: The targets corresponding to the top 30 pathways

| Targets | | | | |
| --- | --- | --- | --- | --- |
| ENGASE | PLA2G2A | AKT1 | HRAS | CTSL |
| NEU3 | PRKACA | MAPK14 | FUCA1 | JUN |
| NEU2 | CHUK | TLR9 | AKR1C3 | PPP1CC |
| NEU4 | HDAC2 | JAK1 | ALOX12 | F2 |
| LDHA | PRKCE | RPS6KB1 | HK2 | ABL1 |
| BCL2L1 | HTR2B | PGK1 | HK1 | MET |
| SERPINE1 | IGF1R | JAK2 | TBXAS1 | IRAK4 |
| PTPN2 | CD38 | CAPN2 | GRM1 | PIK3CB |
| ITGB1 | XPO1 | FLT1 | MGLL | PIK3CG |
| ITGAL | TUBB1 | PDGFRA | CASP3 | PDGFRB |
| PRKCD | MAP2K1 | SRC | HMOX1 | ROCK1 |
| PRKCA | PIK3CA | PTK2B | CASP7 | CDK2 |
| CASP8 | TYK2 | PTGS1 | MAPKAPK2 | IKBKB |
| CASP1 | TRPV1 | BDKRB2 | RPS6KA1 | TRPA1 |
| MMP2 | CCNE2 | HDAC1 | RORC | ALOX5 |
| GRM5 | CCNE1 | FLT3 | CSF1R | STAT3 |
| KDR | MAPK10 | SYK | MAPK1 | NOS2 |
| EPHX2 | CREBBP | CTSK | HTR2C | MDM2 |
| HSP90AA1 | CDC25B | DRD1 | CAMK2D | BTK |
| NOS3 | JAK3 | CCND3 | MAPK9 | MAPK8 |
| PIM1 | ZAP70 | ROCK2 | PRKCB | PDPK1 |
| RPS6KA3 | ADORA1 | GSK3B | PRKCG |  |

Table S2: Content of 16 compounds in 28 batches of QZCs

| Compounds  Batches | Content (*n*=2, mg g^-1^) | | | | | | | | | | | | | | | |
| --- | --- | --- | --- | --- | --- | --- | --- | --- | --- | --- | --- | --- | --- | --- | --- | --- |
|  | Gaa | Pra | Nea | Cha | Cra | Loa | Cor | Log | IaB | IaA | IaC | AnA | Coa | Ost | Col | AsD |
| S1 | 2.831 | 0.1436 | 0.5463 | 1.438 | 0.5297 | 3.837 | 1.395 | 0.7255 | 0.7053 | 0.4376 | 0.6663 | 1.012 | 0.8356 | 1.749 | 0.2278 | 11.22 |
| S2 | 3.479 | 0.1766 | 0.5289 | 1.467 | 0.4993 | 4.172 | 1.890 | 0.7187 | 0.7176 | 0.4849 | 0.7758 | 0.9201 | 0.7822 | 1.620 | 0.2114 | 11.44 |
| S3 | 3.244 | 0.1613 | 0.4604 | 1.574 | 0.4717 | 4.953 | 1.713 | 0.9804 | 0.7217 | 0.4436 | 0.6733 | 1.019 | 0.7512 | 1.613 | 0.2104 | 17.09 |
| S4 | 3.277 | 0.1636 | 0.5921 | 1.521 | 0.5836 | 4.397 | 1.670 | 0.7983 | 0.7910 | 0.5035 | 0.8251 | 0.8886 | 0.7699 | 1.612 | 0.2134 | 12.88 |
| S5 | 3.486 | 0.1672 | 0.5179 | 1.433 | 0.5256 | 4.118 | 1.872 | 0.7487 | 0.7146 | 0.4735 | 0.7475 | 0.8843 | 0.7557 | 1.579 | 0.2039 | 11.88 |
| S6 | 3.779 | 0.1846 | 0.5486 | 1.452 | 0.5475 | 4.059 | 2.020 | 0.7596 | 0.7151 | 0.4851 | 0.7418 | 0.8853 | 0.7487 | 1.588 | 0.2053 | 11.90 |
| S7 | 3.387 | 0.1641 | 0.5510 | 1.491 | 0.5477 | 4.252 | 1.941 | 0.7683 | 0.7576 | 0.4989 | 0.8013 | 0.9142 | 0.7702 | 1.600 | 0.2037 | 11.95 |
| S8 | 3.452 | 0.1718 | 0.5809 | 1.486 | 0.5393 | 4.136 | 2.274 | 0.7168 | 0.8066 | 0.5196 | 0.8388 | 0.9157 | 0.7918 | 1.668 | 0.2171 | 12.29 |
| S9 | 3.068 | 0.1523 | 0.5346 | 1.468 | 0.4961 | 3.831 | 1.681 | 0.7304 | 0.7313 | 0.4723 | 0.7533 | 1.035 | 0.8362 | 1.762 | 0.2292 | 11.01 |
| S10 | 3.415 | 0.1763 | 0.5222 | 1.449 | 0.5052 | 4.120 | 1.837 | 0.7353 | 0.7178 | 0.4821 | 0.7796 | 0.9069 | 0.7729 | 1.626 | 0.2122 | 11.39 |
| S11 | 2.865 | 0.1483 | 0.5595 | 1.498 | 0.5322 | 4.205 | 1.561 | 0.7844 | 0.7510 | 0.4749 | 0.7780 | 1.010 | 0.8217 | 1.714 | 0.2249 | 11.77 |
| S12 | 2.867 | 0.1487 | 0.5054 | 1.609 | 0.4889 | 5.392 | 1.488 | 0.9989 | 0.7254 | 0.4514 | 0.8015 | 1.245 | 0.8377 | 1.774 | 0.2230 | 18.24 |
| S13 | 3.961 | 0.1775 | 0.5348 | 1.388 | 0.5273 | 4.071 | 2.159 | 0.7508 | 0.7724 | 0.4703 | 0.7485 | 1.189 | 0.8811 | 1.749 | 0.2266 | 11.82 |
| S14 | 3.822 | 0.1693 | 0.5574 | 1.418 | 0.5390 | 4.156 | 2.316 | 0.7318 | 0.7876 | 0.4776 | 0.7793 | 1.156 | 0.8916 | 1.737 | 0.2238 | 11.68 |
| S15 | 3.662 | 0.1752 | 0.5157 | 1.496 | 0.5264 | 3.957 | 1.784 | 0.7398 | 0.7306 | 0.4866 | 0.7716 | 1.055 | 0.8259 | 1.777 | 0.2176 | 11.16 |
| S16 | 3.642 | 0.1594 | 0.4928 | 1.354 | 0.4700 | 3.640 | 2.213 | 0.6843 | 0.7191 | 0.4500 | 0.7251 | 1.148 | 0.8601 | 1.713 | 0.2257 | 10.54 |
| S17 | 2.644 | 0.1359 | 0.5152 | 1.497 | 0.5070 | 4.914 | 1.332 | 0.9436 | 0.7267 | 0.4159 | 0.7442 | 1.199 | 0.8095 | 1.720 | 0.2188 | 16.86 |
| S18 | 3.127 | 0.1342 | 0.4468 | 1.420 | 0.4622 | 4.920 | 1.715 | 0.9707 | 0.7113 | 0.3942 | 0.7031 | 0.9140 | 0.7990 | 1.572 | 0.2281 | 15.22 |
| S19 | 3.480 | 0.1507 | 0.5072 | 1.446 | 0.5327 | 4.065 | 1.881 | 0.7655 | 0.7517 | 0.4936 | 0.7760 | 0.8528 | 0.7598 | 1.607 | 0.2105 | 10.98 |
| S20 | 2.695 | 0.1175 | 0.4863 | 1.586 | 0.5338 | 5.472 | 1.839 | 1.003 | 0.7783 | 0.4550 | 0.7849 | 1.166 | 0.8159 | 1.694 | 0.2192 | 15.91 |
| S21 | 4.049 | 0.1422 | 0.4775 | 1.397 | 0.5212 | 3.884 | 2.190 | 0.7492 | 0.8242 | 0.4998 | 0.7851 | 1.052 | 0.8106 | 1.567 | 0.2017 | 11.68 |
| S22 | 2.673 | 0.1646 | 0.4811 | 1.562 | 0.4363 | 5.375 | 0.9905 | 0.9369 | 0.7830 | 0.4522 | 0.7436 | 1.213 | 0.8294 | 1.771 | 0.2203 | 17.18 |
| S23 | 2.913 | 0.1386 | 0.5017 | 1.584 | 0.5919 | 6.008 | 1.777 | 0.9716 | 0.8206 | 0.4847 | 0.8320 | 1.061 | 0.8462 | 1.605 | 0.2148 | 11.72 |
| S24 | 3.301 | 0.1399 | 0.4899 | 1.500 | 0.6689 | 4.674 | 2.034 | 0.7465 | 0.8372 | 0.5321 | 0.7559 | 0.8593 | 0.7892 | 1.663 | 0.2216 | 11.98 |
| S25 | 2.988 | 0.1182 | 0.3662 | 1.487 | 0.5262 | 4.999 | 1.981 | 0.9029 | 0.7433 | 0.4369 | 0.6647 | 0.9047 | 0.8029 | 1.599 | 0.2354 | 16.22 |
| S26 | 3.970 | 0.1677 | 0.4957 | 1.431 | 0.5649 | 3.794 | 2.229 | 0.7098 | 0.7279 | 0.4665 | 0.7024 | 1.036 | 0.8577 | 1.606 | 0.2047 | 9.989 |
| S27 | 2.848 | 0.1394 | 0.5428 | 1.460 | 0.5294 | 3.802 | 1.369 | 0.7111 | 0.7547 | 0.4569 | 0.6495 | 0.9675 | 0.8409 | 1.770 | 0.2359 | 9.652 |
| S28 | 3.687 | 0.1764 | 0.4926 | 1.462 | 0.5257 | 3.802 | 1.755 | 0.7190 | 0.7512 | 0.5023 | 0.7566 | 0.9080 | 0.8267 | 1.738 | 0.2229 | 11.41 |
